# Natto May Alleviate Retinoic Acid-Induced Osteoporosis by Activating Gut Microbiota–Bile Acid Axis and OPG/RANKL Signaling Pathway

**DOI:** 10.3390/nu18121927

**Published:** 2026-06-14

**Authors:** Bimi Zhang, Mubai Sun, Yongfu Liu, Tong Pan, Xuecong Zhang, Yuguang He, Xuetong Gan, Da Li, Xinyu Miao, Zhengyang Luo, Honghong Niu, Mei Hua, Jinghui Wang

**Affiliations:** 1 Institute of Agro-Food Technology, Jilin Academy of Agricultural Science (Northeast Agricultural Research Center of China), Changchun 130033, China; 2 Food and Drug Engineering Institute, Jilin Provincial Institute of Economic Management and Administration, Changchun 130012, China; 3 Jilin Province Agricultural Product Processing Promotion Center, Changchun 130022, China

**Keywords:** natto, osteoporosis, OPG/RANKL signaling pathway, gut microbiota, serum metabolome, bile acid synthesis

## Abstract

Background: Natto, a well-known fermented soybean product beneficial for bone health, remains unclear in its mechanism. Methods: This study investigated its effect on secondary osteoporosis (OP) in mice. Results: Natto significantly inhibited weight loss, bone quality deterioration, and bone morphological damage, and regulated OPG/RANKL pathway protein expression (*p* < 0.05) in OP mice. Analysis of 16S rRNA revealed that natto increased gut microbiota α-diversity and the abundance of *Sutterella*, *Roseburia*, and *Coprococcus*, while reducing harmful bacteria such as *Streptococcus*, *Shigella*, and *Helicobacter*. These microbial changes positively correlated with body weight, bone size, and serum osteogenic metabolism in OP mice. Serum metabolomics showed differential metabolites of the natto group enriched in PPAR signaling and primary bile acid biosynthesis. Verification by mRNA and ELISA indicated that the upregulated liver and circulating PPARα by natto may regulate downstream bile acid pathways, linking gut microbiota to multi-organ metabolic functions. Conclusions: In summary, natto may act on gut microbiota to alleviate bone loss via the “gut microbiota–bile acid–OPG/RANKL” network, targeting multiple organs including gut, liver, and bone. This provides a theoretical basis for natto dietary intervention in osteoporosis prevention through the gut–bone axis.

## 1. Introduction

Osteoporosis (OP) is a systemic bone disease resulting from various factors, characterized by increased bone fragility and susceptibility to fractures. It is reported that osteoporosis affects one-third of women and one-fifth of men worldwide and is a leading cause of disability and mortality among the elderly [1]. Morphologically, osteoporosis is primarily marked by reduced bone mass and deterioration of bone microarchitecture. At the molecular level, the underlying cause is an imbalance in which bone resorption exceeds bone formation. Current pharmacological treatments for osteoporosis, such as estradiol and bisphosphonates, are commonly used to inhibit bone resorption but do not promote bone formation. Moreover, their side effects may lead to excessive suppression of bone turnover and complications such as atypical fractures [2]. As the global population continues to age rapidly, research into the prevention and treatment of osteoporosis is of great significance for improving public health. Dietary intervention is considered the most acceptable long-term strategy for the prevention and management of osteoporosis [3].

Natto is produced by fermenting cooked soybeans with Bacillus natto (BN). It is rich in key bioactive components, including soy isoflavones, nattokinase, and vitamin K_2_ [4]. Fermentation enables the original soy isoflavone components in soybeans, which are not easily absorbed by the small intestine, to increase their absorption rate in the small intestine by combining with sugar [5]. This beneficial component, known as phytoestrogen, can reduce the occurrence of osteoporosis caused by decreased estrogen levels, enhance the activity of osteoblasts (OB), increase bone formation, inhibit the differentiation and bone resorption of osteoclasts (OC), and thereby maintain the normal balance of bone metabolism [6,7]. Additionally, the vitamin K_2_ produced by natto is considered a safe and effective supplement for preventing bone loss [8,9].

Osteoporosis in mammals results from an imbalance in the metabolic activity between OB and OC within the bone matrix. Heightened osteoclast activity coupled with reduced osteoblast function leads to bone resorption exceeding bone formation, ultimately causing decreased bone mass and deterioration of bone microstructure [10]. Consumption of natto has been shown to preserve bone mass through molecular mechanisms distinct from those of exercise, alleviating bone loss in ovariectomized rats [11]. Specifically, soy isoflavones influence the key bone remodeling pathway—the osteoprotegerin/receptor activator of nuclear factor-κB ligand (OPG/RANKL) system—through multiple mechanisms. They reduce levels of RANKL and increase OPG levels, thereby decreasing serum markers of bone resorption, improving bone metabolism, and reducing bone loss [12]. Additionally, vitamin K_2_, abundant in natto, has been demonstrated to promote bone formation and inhibit bone resorption by increasing alkaline phosphatase activity, stimulating osteoblast differentiation, regulating bone extracellular matrix mineralization, upregulating bone marker gene expression, and suppressing osteoclastogenesis [13].

More importantly, as a fermented soy product beloved by people in Asian countries, natto has also been proven to have the effect of increasing bone density and promoting calcium absorption in elderly men, elderly women, and especially postmenopausal women [14]. However, these data based on human medical cohort studies still lack further mechanism analysis. Bone formation relies on the precise coordinated metabolic regulation of systems including the liver, muscles, adipose tissue, and pancreatic β cells. Integration of multidisciplinary research on immunology and inflammation helps to evolve our related understanding [15]. For instance, the differentiation and activation of OC are promoted by various factors including inflammatory cytokines and chemokines produced by immune cells [16]. In recent years, with the continuous in-depth research on the gut microbiota, it has been discovered that gut microorganisms can regulate bone metabolism through the endocrine system, the immune system, and their metabolites—a concept known as the “gut–bone” axis [17]. As a fermented soy product with a long history of consumption, the regulatory effects of natto on bone, intestine, and metabolism are widely recognized. However, whether its beneficial effects on these two major organs are interconnected, and the specific mechanisms underlying such a relationship, remain unclear. This knowledge gap has hindered the full utilization of natto’s valuable osteoprotective and health-promoting properties. Therefore, to elucidate the mechanism by which natto improves osteoporosis, this study established a mouse model of osteoporosis via intragastric administration of retinoic acid. The effects of natto supplementation on bone tissue structure, bone metabolic pathways, gut microbiota, and serum metabolic profiles were investigated, aiming to provide a theoretical basis for the development of natto-based functional foods that enhance bone health.

## 2. Materials and Methods

### 2.1. Feed Preparation

To prepare the feed, 10% (*v*/*w*) of the laboratory self-sieved strain *Bacillus natto* JLCC513 fermentation broth was added to steamed soybeans. The mixture was left to ferment at 37 °C in a fermentation box for 24 h to produce natto. The natto was then freeze-dried and ground into powder for storage (with an effective viable bacterial count of 1.0 × 10^7^ CFU/g and a nattokinase content of 2019.77 IU/g). The ordinary maintenance feed was purchased from Liaoning Changsheng Biotech Co. (Benxi, China). Then, 2.5% *w*/*w* of natto freeze-dried powder was added to the ordinary maintenance feed to make natto feed. This was produced by Jiangsu XieTong Medical Biotechnology Engineering Co., Ltd. (production license: Su Si Zhi (2019) 01008. Yangzhou, China). The dosage of natto added is based on the conversion from the daily consumption of 150 g natto (i.e., 15 g of dried natto powder) by an adult weighing 60 kg, and the daily food intake of 3 g for 30 g ICR mice.

### 2.2. Animal Experiment

Eighteen SPF-grade healthy female ICR mice, weighing 27–30 g, were purchased from Liaoning Changsheng Biotech Co. (License No.: SCXK (Liao) 2020-0001). All animal experiments were conducted in accordance with the guidelines established by the Laboratory Animal Management and Welfare Ethics Committee of the Institute of Agricultural Product Processing of Jilin Academy of Agricultural Sciences (authorization number: LAMECJAAS-2023-021-03).

After 7 days of adaptive feeding, the mice were randomly divided into three groups (n = 6): control group (ND), model group (OP), and natto intervention group (Natto). During the experiment (21 days), all mice had free access to water and food. The ND and OP group were fed with basic maintenance feed, while the Natto group was fed with natto feed. At the end of the experiment, the OP and Natto group were intragastrically administered retinoic acid suspension (dissolved in corn oil, YuanYe, S30976) with 105 mg/kg/day for 1–7 days and 75 mg/kg/day for 8–21 days, and the ND group was given the same volume of normal saline. One day before the end of the modeling and administration, fecal samples were collected from each group of mice and stored at −80 °C. Subsequently, the mice were fasted but not dehydrated for 12 h, and their body weight was measured before blood collection. Blood samples were centrifuged at 4 °C, 3000 r/min for 10 min, and the upper serum was separated and stored at −80 °C. The mice were then decerebrated, and their left and right femoral and tibial bones were scraped off on ice, with the extra muscles and mucosal tissues removed from both sides. The left bone was frozen at −80 °C for analysis of bone characteristics and other tests, and the right bone was immersed in 4% paraformaldehyde for tissue sectioning observation.

### 2.3. Bone Mechanical Properties Analysis

The left bone of each mouse was immersed in 4°C physiological saline for 4 h, and the bone diameter and length (the midpoint of the bone is the bone diameter, and the distance from the proximal end of the femur to the distal end of the femur is the length of the femur) were measured. The femur and tibia were weighed using an electronic balance, and the TA-XTplus100C Texture analyzer (Godalming, UK) were used to conduct three-point bending tests on the femur and tibia. The femur was attached to the fixture, with a distance between the two ends of support of 7 mm, and then the probe was descended at a speed of 1 mm/s until it reached the point where the femur and tibia break. The force measured at this time was the fracture failure force.

### 2.4. Serum Bone Metabolism Indicators Analysis

Mouse alkaline phosphatase (ALP, RD-RX20406J), mouse tartrate-resistant acid phosphatase (TRAP, RD-RX20440J), mouse osteocalcin (OCN, RD-RX20334J), and mouse *N*-terminal propeptide of type I procollagen (PINP, RD-RX20072J) ELISA kits were purchased from Ruida Henghui Technology Development Co., Ltd. (Beijing, China). Blood phosphorus (P, BC1650) concentration detection kits were obtained from Solarbio Science & Technology Co., Ltd. (Beijing, China). The above indicators were measured according to the instructions provided in the kit manuals.

### 2.5. Histological Observation

Right femurs were collected and fixed in 4% paraformaldehyde solution at 4 °C for 48 h, followed by decalcification in 15% EDTA solution (pH 8.0) for 4 weeks. The decalcification solution was replaced every three days. After decalcification, the bone tissues were dehydrated, embedded in paraffin, and sectioned. Hematoxylin-eosin (HE) and Masson staining were performed, and the sections were observed and photographed under a light microscope. Image Pro Plus 6.0 software was used for quantitative analysis of the images.

### 2.6. Immunohistochemical Analysis

Decalcified samples were dehydrated through a graded alcohol series, embedded in paraffin, and sectioned at a thickness of 4 μm. Immunohistochemical (IHC) staining was performed using an immunohistochemistry kit (Servicebio, G1212, Wuhan, China). The sections were observed under a light microscope for result interpretation. Hematoxylin-stained nuclei appeared blue, while DAB-stained positive expression appeared brownish-yellow. Observations and photographs were taken under a light microscope, and Image Pro Plus software was used for quantitative analysis of the images.

### 2.7. Western Blot (WB) Analysis

The femoral tissue (30 mg) was homogenized by adding RIPA lysate and to measure the protein quantification with the BCA Protein Kit (Beyotime, P0010, Shanghai, China). Protein extract (5 μg/μL) was mixed with loading buffer (Servicebio, G2075-1ML, Wuhan, China) and heated in a boiling water bath for 10 min. Denatured proteins were separated by SDS-PAGE and transferred to a PVDF membrane (Millipore, ISEQ00010, Shanghai, China). The membrane was blocked with a protein-free blocking solution (Servicebio, G2052-500ML, Wuhan, China) for 5 min at room temperature, incubated overnight at 4 °C with the primary antibody (OPG Rabbit pAb, YT3466, RANKL Rabbit pAb, YT5404, Immunoway Biotechnology Co., Ltd., Suzhou, China), and subsequently incubated with a horseradish peroxidase-conjugated secondary antibody. Finally, a SuperECL Plus Kit (UElandy, S6009, Suzhou, China) was used to exhibit the band. Protein bands were visualized on a chemiluminescence imaging system (Servicebio, SCG-W3000, Wuhan, China). Band grayscale values were quantified using ImageJ V1.8.0 software and normalized to β-actin (Servicebio, GB15003-100, Wuhan, China).

### 2.8. Gene Expression Analysis

Total RNA was extracted using RNA extraction reagent, ethanol, and other reagents. The concentration and purity of the extracted RNA were measured using a Nanodrop 2000c spectrophotometer (Thermo Fisher Scientific, Waltham, MA, USA). Reverse transcription PCR was performed in a thermal cycler to amplify the DNA, and real-time quantitative PCR was conducted to detect the mRNA expression of peroxisome proliferator-activated receptor alpha (PPARα). The reaction conditions were as follows: pre-denaturation at 95 °C for 30 s, followed by 40 cycles of amplification at 95 °C for 15 s and 60 °C for 30 s. The melting curve was generated by increasing the temperature from 65 °C to 95 °C, with fluorescence signal collected at every 0.5 °C increment. The obtained data were analyzed using the 2^−△△CT^ method for relative quantification. The primer sequences were as follows: GAPDH: 5′-CCTCGTCCCGTAGACAAAATG-3′, 5′-TGAGGTCAATGAAGGGGTCGT-3′; PPARα: 5′-TGATGCAGATCGTGGACCTCT-3′, 5′-CGAGTGGTAGGTTGGTATCG-3′.

### 2.9. High Throughput 16S rRNA Gene Diversity in Gut Microbiota

Total genomic DNA from enteric bacteria was extracted using the QIAamp Fast DNA Extraction Kit (QIAGEN, Shanghai, China), following the manufacturer’s instructions. Sample concentration and purity were assessed using a NanoDrop 2000c spectrophotometer (Thermo Fisher Scientific, Waltham, MA, USA) and verified by agarose gel electrophoresis. The V3-V4 region of the bacterial 16S rRNA gene was amplified by PCR using a forward primer (5′-ACTCCTACGGGGAGGCAGCA-3′) and a reverse primer (5′-TCGGACTACHVGGGTWTCTAAT-3′). The PCR reaction mixture (20 µL) contained 5 × TransStart FastPfu PCR Buffer (4 µL), dNTPs (2.5 mmol/L, 2 µL), TransStart FastPfu DNA Polymerase (2.5 U/µL, 0.5 µL), forward and reverse primers (10 µmol/L, 0.5 µL each), template DNA (2 µL), and ddH_2_O (10.5 µL). PCR cycling conditions were as follows: initial denaturation at 94 °C for 4 min; 30 cycles of 94 °C for 30 s, 50 °C for 30 s, and 72 °C for 30 s; final extension at 72 °C for 5 min. Amplified products were quantified using the PicoGreen dsDNA Quantification Kit (Invitrogen, Carlsbad, CA, USA).

Pooled amplified sequences were subjected to paired-end sequencing on the Illumina NovaSeq 6000 System with the MiSeq Reagent Kit v3 (Shanghai Personal Biotechnology Co., Shanghai, China). Sequencing data were processed through the Quantitative Insights Into Microbial Ecology (QIIME 2.0, 2019.4) pipeline. Quality control steps, including de-priming, quality filtering, denoising, splicing, and chimera removal, were performed with the DADA2 method. Denoised high-quality sequences generated by DADA2 were clustered into non-singleton amplicon sequence variants (ASVs) using UCLUST (Release 13.8, https://greengenes.lbl.gov/Download/, accessed on 16 April 2026). ASVs with an abundance below 0.001% of total sequences were discarded from all samples. Representative sequences were selected from the ASVs using default parameters and taxonomically classified using the BLAST (qiime2/sklearn) method in the Greengenes database on a rank-by-rank basis. Venn diagram was generated to visualize the shared and unique ASVs among samples or groups using R script (v4.1.0) “VennDiagram (v1.7.4)”, based on the occurrence of ASVs across samples/groups regardless of their relative abundance. R script (v4.1.0) was used to calculate the clustering results of each sample and each taxonomic unit, and present the heat map of the species composition of each group in the form of an interactive graph. Using the absolute abundance table of taxonomic units at the phyla, class, order, family, and genus generated by the unextracted ASV table, the “classify_samples_ncv” function in q2-sample-classifier was called to conduct random forest analysis to infer the representative species strains of each group.

### 2.10. Serum Metabolomics Analysis

#### 2.10.1. Liquid Chromatography-Mass Spectrometry (LC-MS) Conditions

Samples were analyzed using a Thermo Vanquish ultra-high performance liquid chromatography (UPLC) system (Thermo Fisher Scientific, Waltham, MA, USA) equipped with an ACQUITY UPLC^®^ HSS T3 column (2.1 × 100 mm, 1.8 µm) (Waters, Milford, MA, USA). The flow rate was 0.3 mL/min, the column temperature was maintained at 40 °C, and the injection volume was 2 µL. In positive ion mode, the mobile phase consisted of 0.1% formic acid in acetonitrile (B1) and 0.1% formic acid in water (A1). The gradient elution program was as follows: 0–1 min, 8% B1; 1–8 min, 8–98% B1; 8–10 min, 98% B1; 10–10.1 min, 98–8% B1; 10.1–12 min, 8% B1. In negative ion mode, the mobile phase consisted of acetonitrile (B2) and 5 mM ammonium formate in water (A2). The gradient elution program was: 0–1 min, 8% B2; 1–8 min, 8–98% B2; 8–10 min, 98% B2; 10–10.1 min, 98–8% B2; 10.1–12 min, 8% B2.

#### 2.10.2. Data Processing Methods

The raw data were firstly converted to mzXML format by MSConvert in ProteoWizard software package (v3.0.8789) and processed using R XCMS (v3.12.0) for feature detection, retention time correction, and alignment. Key parameters settings were set as follows: ppm = 15, peakwidth = c(5, 30), mzdiff = 0.01, method = centWave. Then, the data was corrected by the area normalization method to eliminate systematic errors.

The metabolites were identified by accuracy mass and MS/MS data which were matched with HMDB (https://hmdb.ca/), massbank (https://massbank.eu/MassBank/, accessed on 16 April 2026), KEGG (https://www.kegg.jp/), LipidMaps (http://www.lipidmaps.org), mzcloud (https://www.mzcloud.org) and the metabolite database built by Personal Biotechnology Co., (Shanghai, China). The molecular weight of metabolites was determined according to the m/z (mass-to-charge ratio) of parent ions in MS data. Molecular formula was predicted by ppm (parts/million) and adduction, then matched with the database. At the same time, the MS/MS data from quantitative table of MS/MS data, were matched with the fragment ions and other information of each metabolite in the database, so as to realize the MS/MS identification of metabolites.

Two different multivariate statistical analysis models, unsupervised and supervised, were applied to discriminate the groups (PCA; PLS-DA; OPLS-DA) by R ropls (v1.22.0) package. The statistical significance of the *p* value was determined by statistical test between groups. Finally, Variable Importance in Projection (VIP, OPLS-DA variable projection importance) and Fold Change (FC, multiple of difference between groups) were combined with the *p* value to screen biomarker metabolites. By default, when the *p* value < 0.05 and the VIP value > 1, the metabolites were considered to have significant differential expression.

Pathway enrichment analysis used the hypergeometric distribution enrichment analysis method to perform functional pathway enrichment and topological analysis of metabolites. The identified metabolites in metabolomics were then mapped to the KEGG pathway for biological interpretation of higher-level systemic functions. The metabolites and corresponding pathways were visualized using KEGG Mapper tool.

### 2.11. Statistical Analysis

All results are expressed as means ± standard deviation, significant differences at *p* < 0.05 (*), and highly significant differences at *p* < 0.01 (** or ##). Graphpad Prism 9.0 was used for drawing, ImageJ was used for area calculation, and IBM SPSS Statistics 27.0 was used for statistical analysis. Before performing ANOVA, the homogeneity of variances was assessed using Levene’s test. The one-way ANOVA method was used to evaluate the treatment effects of each outcome, and least significant difference (LSD) method was selected for post hoc analysis to compare the means between groups. There were no adverse events during the experiment (e.g., attacks or bites, drug allergies, abnormal deaths), so no animals or data points were excluded from the study during the analysis process.

For analysis of 16S rRNA sequencing data, differential abundance analysis at the genus level was performed using SPSS Statistics 27.0 The screening criterion for differential bacterial taxa was based on adjusted *p* value (*p*adjust < 0.05) to define statistical significance. The Padjust was calculated using the Benjamini–Hochberg false discovery rate (FDR) correction method to control for multiple hypothesis testing. 

For serum metabolomics data, both univariate and multivariate statistical analyses were combined to screen for differential metabolites between groups. Univariate analysis was performed using Student‘s *t*-test (two-tailed, unpaired), with *p* value < 0.05 as the screening threshold. Multivariate analysis was conducted using OPLS-DA, with VIP > 1 as the selection criterion for differential metabolites. Metabolites meeting both OPLS-DA VIP > 1 and *p* value < 0.05 were considered as statistically significant differential metabolites. Subsequently, KEGG pathway enrichment analysis was performed on the identified differential metabolites using a hypergeometric distribution-based enrichment method, with *p* value < 0.05 considered statistically significant.

## 3. Results and Analysis

### 3.1. Effect of Natto on Body Weight in OP Mice

**Figure 1:**
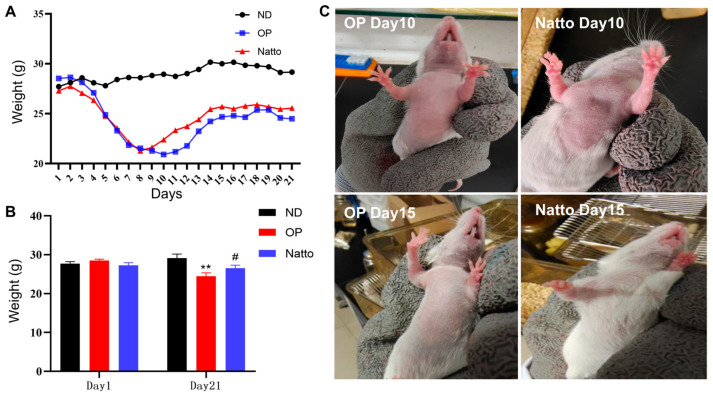
Effect of natto on OP mice weight and the appearance (n = 6). (**A**) Curve of mouse body weight fluctuations during the experiment. (**B**) The body weight difference of mice before and after the experiment. (**C**) Mice appearance (focus on the lower abdomen). ND group vs. OP group (**, *p* < 0.01), OP group vs. Natto group (#, *p* < 0.05).

As shown in Figure 1, during the 21-day experimental period, the administration of 105 mg/kg retinoic acid in the first 7 days for modeling resulted in a rapid decrease in body weight, sluggish movement, and poor mental state in both the OP group and the Natto group. Therefore, from day 8 onward, the retinoic acid dose was adjusted to 75 mg/kg. Subsequently, the body weight of mice in the Natto group increased significantly, and by the end of the experiment, it was markedly higher than that of the OP group (*p* < 0.05), accompanied by more active behavior. Notably, retinoic acid also caused abdominal hair loss in mice, a phenomenon that was significantly alleviated in the Natto group. These findings suggest that natto can effectively counteract retinoic acid-induced symptoms such as weight loss, hair loss, and reduced activity in mice.

### 3.2. Effect of Natto on Femur and Tibia Weight, Length, and Diameter in OP Mice

Bone weight, length, and diameter reflect the level of skeletal development in mice [10]. Compared with the ND group, the femoral and tibial length, diameter, and weight were significantly decreased in the OP group (*p* < 0.05, Table 1). Compared with the OP group, the Natto group exhibited significantly increased femoral length, diameter, and weight, as well as tibial diameter and weight (*p* < 0.05), approaching or reaching the levels of the normal group (Table 1).

### 3.3. Effect of Natto on Fracture Force and Serum Bone Metabolism Markers in OP Mice

Osteoporosis leads to changes in bone microstructure, increased bone fragility, thinning and reduction of bone trabeculae, widening of intertrabecular spaces, and increased fracture susceptibility. Bone fracture force can be used to assess the difficulty of inducing bone fractures [18]. As shown in Table 2, compared with the ND group, the femoral fracture force in the OP group decreased by 53.33%, and the tibial fracture force decreased by 51.41% (*p* < 0.01). In contrast, compared with the OP group, the Natto group exhibited an 83.53% increase in femoral fracture force (*p* < 0.01) and an 80.44% increase in tibial fracture force (*p* < 0.01). These results indicate that natto supplementation can effectively improve the biomechanical strength of the femur and tibia in OP mice.

The main organic component of bone, osteoid (which undergoes mineralization to form hydroxyapatite crystals), is synthesized by OB. Alkaline phosphatase (ALP), produced by matrix vesicles secreted by OBs, plays a role in bone calcification and serves as a marker of osteoblast maturation and activity, representing a typical indicator of bone formation [19,20]. Tartrate-resistant acid phosphatase (TRAP), produced by OC, is responsible for decomposing organic components and participates in the degradation of solid calcium phosphate minerals in the bone matrix. Procollagen type I *N*-terminal propeptide (PINP), derived from type I collagen—the most abundant collagen expressed by proliferating OB—reflects the intensity of bone formation. Osteocalcin (OCN), expressed by OB during the mineralization process, plays a role in mineral binding [19]. Phosphorus (P) is a major component of the inorganic bone matrix; excessive loss of inorganic substances from bone into the bloodstream can lead to osteoporosis [21]. As shown in Table 2, compared with the ND group, both ALP and TRAP levels were significantly increased in the OP group (*p* < 0.05). Compared with the OP group, the Natto group showed a 26.14% reduction in TRAP (*p* < 0.05) and a 36.84% reduction in ALP (*p* < 0.05). Compared with the ND group, the levels of PINP, OCN, and blood phosphorus were significantly decreased in the OP group (*p* < 0.05). Compared with the OP group, the Natto group exhibited increases in PINP, OCN, and P to 3.14 times, 2.16 times, and 1.34 times the OP group levels, respectively (*p* < 0.05), approaching or even exceeding the levels observed in the ND group. Therefore, Natto may ameliorate osteoporosis by enhancing bone formation, reducing bone resorption, and promoting bone mineral binding.

### 3.4. Effect of Natto on Bone Tissue Structure and Metabolism Protein Expression Levels in OP Mice

**Figure 2:**
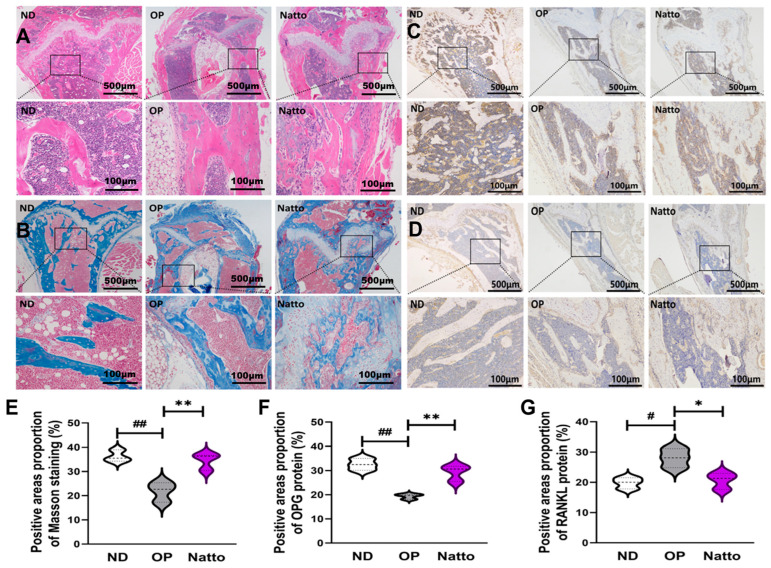
Femoral HE ((**A**) 40× and 100×) and Masson ((**B**) 40× and 100×) staining, IHC staining for OPG ((**C**) 40× and 100×) and RANKL protein ((**D**) 40× and 100×), quantitative analysis of Masson staining positive area (**E**), quantitative analysis of IHC protein positive area for OPG (**F**), and RANKL (**G**). The different colors in the violin plot represent different experimental groups. The three dotted lines, from top to bottom, respectively represent: the upper quartile Q3 value, the median value, and the lower quartile Q1 value. ND group vs. OP group (#, *p* < 0.05; ##, *p* < 0.01), OP group vs. Natto group (*, *p* <0.05; **, *p* < 0.01).

Trabecular bone microstructure and bone morphometric parameters are generally regarded as ideal indicators for predicting bone loss and deterioration of bone structure, and are commonly used to assess the success and severity of osteoporosis models. Femoral HE staining (Figure 2A) revealed that, compared with the ND group, the OP group exhibited sparse trabecular bone structure with uneven thickness, bone marrow edema, increased OC, and extensive adipogenesis. Compared with the OP group, the Natto group showed significantly increased trabecular bone with a more orderly and compact arrangement, abundant osteoid structures with uniform thickness along the trabecular edges, a patent bone marrow cavity, thickened and smooth cortical bone, and no obvious adipocytes. Femoral Masson staining (Figure 2B) showed that, compared with the ND group, the OP group exhibited significant damage to bone soft tissue, with a marked decrease in the area of bone collagen fibers of 21.77% (*p* < 0.01). In contrast, compared with the OP group, the Natto group demonstrated a significantly increased femoral collagen fiber area of 34.50% (*p* < 0.01, Figure 2E).

Osteoprotegerin (OPG) and receptor activator of nuclear factor-κB ligand (RANKL) are members of the tumor necrosis factor superfamily and are important factors involved in the regulation of bone homeostasis. OPG inhibits osteoclast differentiation and maturation, prevents osteoporosis, and increases trabecular bone density; RANKL promotes osteoclast differentiation and maturation [22]. Immunohistochemical results (Figure 2C,D) showed that the positive area for OPG protein immunohistochemical staining in the Natto group (30.85%) was significantly higher than that in the OP group (19.35%) (*p* < 0.01, Figure 2F). The positive area for RANKL protein immunohistochemical staining in the Natto group (20.56%) was significantly lower than that in the OP group (26.03%) (*p* < 0.05, Figure 2G), approaching the level of the ND group. These results indicate that natto exerts an inhibitory effect on osteoclast differentiation by regulating the OPG/RANKL signaling pathway, thereby increasing trabecular bone and bone collagen and maintaining bone structural stability.

### 3.5. Effect of Natto on Bone Metabolism Pathways in OP Mice

**Figure 3:**
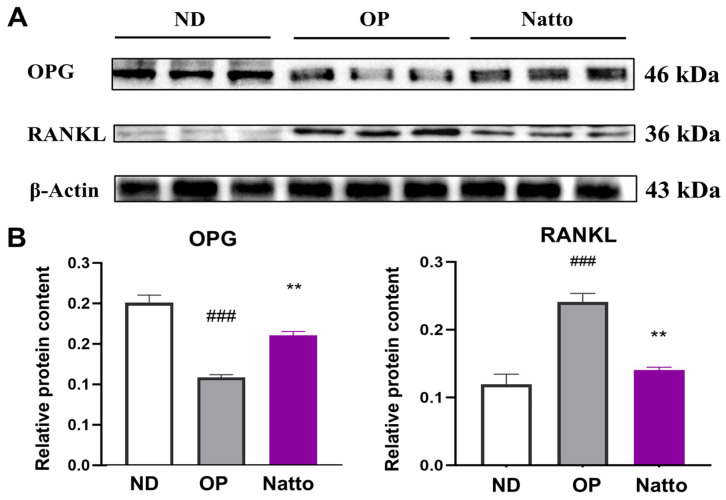
Representative Western blot images of mice OPG and RANKL protein (**A**) and their quantitative analysis (**B**). ND group vs. OP group (###, *p* < 0.001), OP group vs. Natto group (**, *p* < 0.01).

Further Western Blot (WB) analysis confirmed the regulatory effect of natto on the OPG/RANKL pathway (Figure 3), demonstrating that it effectively promoted the expression of OPG protein (*p* < 0.01) and inhibited the expression of RANKL protein (*p* < 0.01) in OP mice.

### 3.6. Effect of Natto on the Diversity and Structure of Gut Microbiota in OP Mice

#### 3.6.1. Changes in Gut Microbiota Diversity

**Figure 4:**
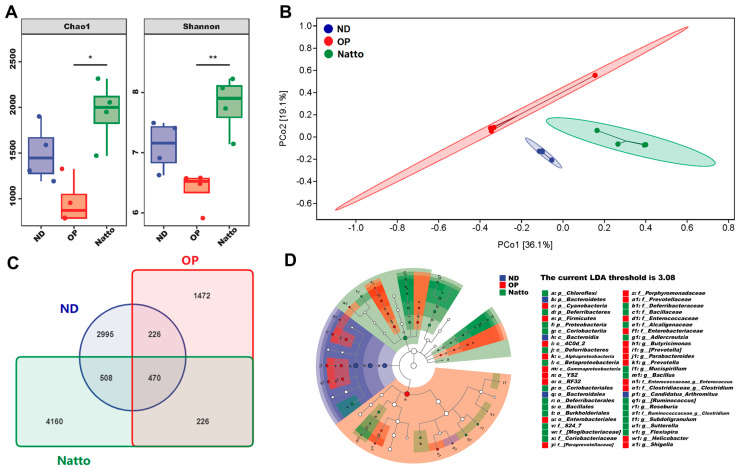
Effect of natto on the Diversity and Structure of Gut Microbiota in OP Mice (n = 4). (**A**) Alpha diversity analysis of mouse gut microbiota; (**B**) Beta diversity analysis of mouse gut microbiota; (**C**) Venn plot of intestinal ASVs; (**D**) Intestinal microbiota LEfSe (LDA Effect Size) analysis of evolutionary branch diagram. OP group vs. Natto group (*, *p* < 0.05; **, *p* < 0.01).

Natto exerted a beneficial effect on the diversity of the gut microbiota in OP mice (Figure 4). Alpha diversity analysis (Figure 4A) showed that retinoic acid disrupted the balance of the gut microbiota in mice, reducing both community richness (Chao1) and diversity (Shannon). Following dietary intervention, the Natto group exhibited significantly higher Chao1 (*p* < 0.05) and Shannon (*p* < 0.01) indices compared to the OP group. Beta diversity analysis using PCoA (Figure 4B) revealed distinct community separation between the OP and the ND group, while the Natto group showed a community structure distribution closer to that of the ND group. The Venn diagram of the gut microbiota (Figure 4C) indicated that, compared with the ND group, the number of unique ASVs in the OP group decreased from 2995 to 1472, a reduction of 50.85%, with only 226 ASVs shared between the two groups. However, after dietary intervention, the number of unique ASVs in the Natto group recovered to 4160, which was 2.83 times that of the OP group’s unique ASVs. The number of ASVs shared between the Natto group and the ND group reached 508, which was 2.25 times the number shared between the ND group and the OP group. Linear discriminant analysis effect size (LEfSe) analysis of the gut microbiota (Figure 4D) also demonstrated that the Natto group (green) and the OP group (red) began to branch off near the base of the cladogram, indicating substantial differences in phylogenetic relationships between the microbial communities of the two groups.

#### 3.6.2. Taxonomic Composition Analysis of Gut Microbiota and Identification of Differential Species Between Groups

**Figure 5:**
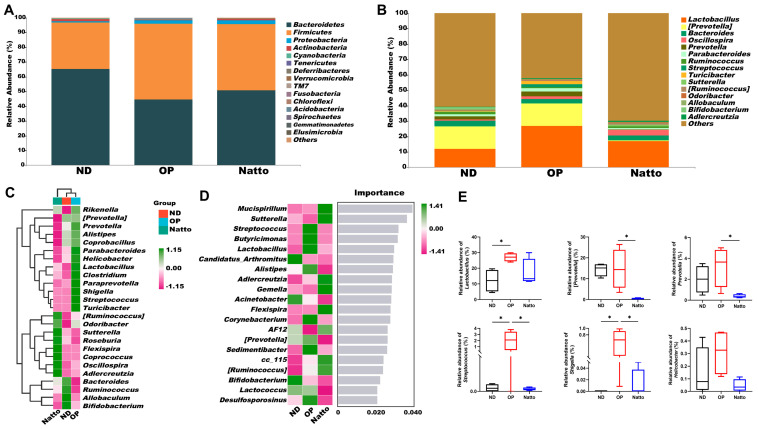
Analysis of the taxonomic composition of mice gut microbiota at the phylum (**A**) and genus (**B**,**E**) levels, composition heat map (**C**) and analysis of random forest model (**D**) at the genus level. ND group vs. OP group, or OP group vs. Natto group (*, *p* < 0.05).

Analysis of taxonomic composition at the phylum and genus levels revealed that, at the phylum level (Figure 5A), the gut microbiota in all groups were primarily dominated by *Firmicutes* and *Bacteroidota*. The relative abundance of *Bacteroidota* in the OP group decreased from 65.33% in the ND group to 47.40%, while the relative abundance of *Firmicutes* increased from 31.71% in the ND group to 48.78%. In the Natto group, the abundance of *Bacteroidota* recovered to 50.96%, and the abundance of *Firmicutes* recovered to 44.67%. Further analysis at the genus level (Figure 5B,C) showed that the OP group exhibited higher abundances of *Helicobacter*, *Lactobacillus*, *Paraprevotella*, *Shigella*, and *Streptococcus*. In contrast, the Natto group showed higher abundances of genera such as *Sutterella*, *Roseburia*, *Flexispira*, *Coprococcus*, and *Oscillospira*. The random forest model (Figure 5D) indicated that potentially opportunistic pathogenic genera such as *Streptococcus* were present in higher abundance and served as signature species in the OP group. In the Natto group, the two most abundant and important genera were *Mucispirillum* and *Sutterella*. Quantitative analysis (Figure 5E) revealed that the Natto group significantly reduced the abundances of *Prevotella*, [*Prevotella*], *Streptococcus*, and *Shigella* (*p* < 0.05) compared to the OP group, and also exhibited a significant inhibitory effect on the abundance of *Helicobacter*.

### 3.7. Effect of Natto on the Serum Non-Target Metabolomics of OP Mice

**Figure 6:**
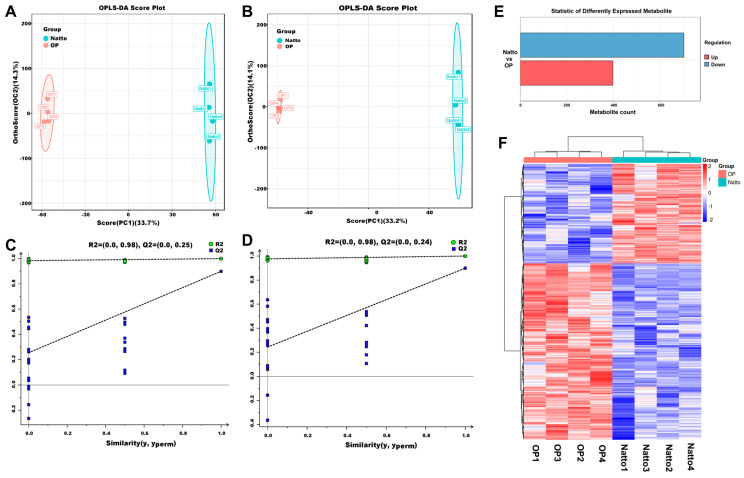
OPLS-DA score plot of the OP and Natto groups in metabolomic analysis (n = 4). Positive ion mode (**A**) and negative ion mode (**B**) of OPLS-DA score plots. Positive ion mode (**C**) and negative ion mode (**D**) of OPLS-DA permutation test plots. (**E**) Histogram of differential metabolites. In the figure, red represents the number of upregulated metabolites, and blue represents the number of downregulated metabolites. (**F**) Cluster heatmap of differential metabolites. A redder color indicates higher expression levels, while a bluer color indicates lower expression levels. Metabolite names are not displayed as the number of metabolites exceeds 150.

To investigate the effect of natto on the serum metabolic profile of OP mice, we employed LC-MS to analyze the differences in serum metabolites between the OP group and the Natto group. As shown in Figure 6, samples from the OP group and Natto group were clearly separated in both positive ion mode (Figure 6A) and negative ion mode (Figure 6B), indicating significant differences between the two groups. The Orthogonal Projections to Latent Structures Discriminant Analysis (OPLS-DA) permutation test plots (Figure 6C,D) confirmed the reliability of the models for both ion modes. Primary differential metabolites were screened based on *p* value and VIP scores. Further identification of secondary metabolites was performed by searching and comparing with spectral databases including HMDB, MassBank, LipidMaps, mzCloud, and KEGG. The qualitative identification of secondary metabolites (Figure 6E,F) revealed a total of 1098 differential metabolites, among which 397 were upregulated (red bars) and 701 were downregulated (blue bars) in the Natto group compared to the OP group.

**Figure 7:**
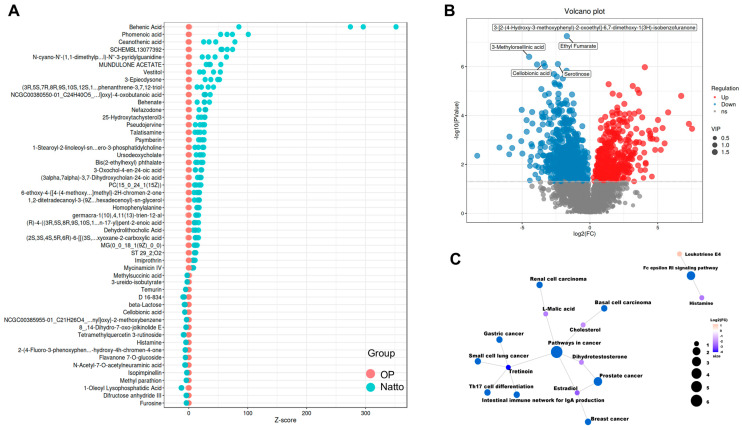
Serum differential metabolites’ Z-scores and enrichment analysis between the OP group and the Natto group. Z-score score plot (**A**). The closer to the right, the higher the relative content of the current metabolite in this sample; the closer to the left, the lower the content of the current metabolite. The volcano plot of the top 5 differentially expressed metabolites by *p* value (**B**) and their enriched KEGG pathways (**C**). The size of the pathway points in C represents the number of metabolites connected to it; the more connected the metabolites, the larger the point. The color of the metabolite points represents the magnitude of log2(FC) value; red indicates up-regulation of difference, blue indicates down-regulation of difference, and the darker the color, the greater the degree of difference. N-cyano-N′-(1,1-dimethylp…l)-N″-3-pyridylguanidine means N-cyano-N′-(1,1-dimethylpropyl)-N″-3-pyridylguanidine; (3R,5S,7R,8R,9S,10S,12S,1…phenanthrene-3,7,12-triol means (3R,5S,7R,8R,9S,10S,12S,13R,14S,17R)-17-((2R)-6,7-dihydroxy-6-methylheptan-2-yl)-10,13-dimethylhexadecahydro-1H-cyclopenta[a]phenanthrene-3,7,12-triol; NCGC00380550-01_C24H40O5…l]oxy}-4-oxobutanoic acid means NCGC00380550-01_C24H40O5_4-{[5-(6-Hydroxy-5,5,8a-trimethyl-2-methylenedecahydro-1-naphthalenyl)-3-methylpentyl]oxy}-4-oxobutanoic acid; 1-Stearoyl-2-linoleoyl-sn…ero-3-phosphatidylcholine means 1-Stearoyl-2-linoleoyl-sn-glycero-3-phosphatidylcholine; 6-ethoxy-4-{[4-(4-methoxy…]methyl}-2H-chromen-2-one means 6-ethoxy-4-{[4-(4-methoxybenzyl)piperazin-1-yl]methyl}-2H-chromen-2-one; 1,2-ditetradecanoyl-3-(9Z…hexadecenoyl)-sn-glycerol means 1,2-ditetradecanoyl-3-(9Z-hexadecenoyl)-sn-glycerol; (R)-4-((3R,5S,8R,9S,10S…n-17-yl)pent-2-enoic acid means (R)-4-((3R,5S,8R,9S,10S,13R,14S,17R)-3-hydroxy-10,13-dimethyl-7-oxohexadecahydro-1H-cyclopenta[a]phenanthren-17-yl)pent-2-enoic acid; (2S,3S,4S,5R,6R)-6-[[(3S,…xyoxane-2-carboxylic acid means (2S,3S,4S,5R,6R)-6-[[(3S,4S,4aR,6aR,6bS,8aR,9S,12aS,14aR,14bR)-9-hydroxy-4-(hydroxymethyl)-4,6a,6b,8a,11,11,14b-heptamethyl-1,2,3,4a,5,6,7,8,9,10,12,12a,14,14a-tetradecahydropicen-3-yl]oxy]-5-[(2S,3R,4S,5S)-4,5-dihydroxy-3-[(2S,3R,4R,5R,6S)-3,4,5-trihydroxy-6-methyloxan-2-yl]oxyoxan-2-yl]oxy-3,4-dihydroxyoxane-2-carboxylic acid; NCGC00385955-01_C21H26O4_4…nyl]oxy}-2-methoxybenzene means NCGC00385955-01_C21H26O4_4-Allyl-1-{[1-(3,4-dimethoxyphenyl)-2-propanyl]oxy}-2-methoxybenzene; 2-(4-Fluoro-3-phenoxyphen…hydroxy-4h-chromen-4-one means 2-(4-Fluoro-3-phenoxyphenyl)-3-hydroxy-4h-chromen-4-one.

Based on the relative quantification of metabolites, Z-scores (standard scores) were calculated. As shown in Figure 7A, among the top 100 differential metabolites ranked by *p* value, 33 were significantly elevated in the Natto group. The top 5 differential metabolites ranked by *p* value (Figure 7B) and their enriched KEGG signaling pathways (Figure 7C) revealed that natto primarily downregulated differential metabolites associated with cancers, including hepatocellular carcinoma, gastric cancer, small cell lung cancer, and prostate cancer.

**Figure 8:**
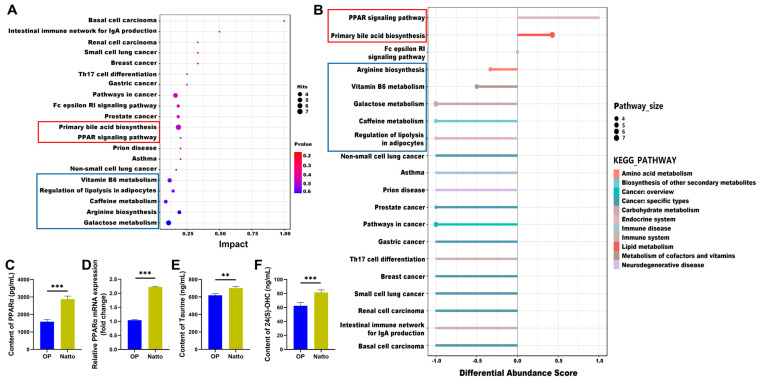
KEGG enrichment analysis of differential metabolites and validation of key substance contents. (**A**) Bubble chart of KEGG metabolic pathway enrichment impact factors for differential metabolites, with the size of the points representing the number of metabolites corresponding to the pathway. The color is related to the *p* value; the redder the color, the smaller the *p* value; the bluer the color, the larger the *p* value. (**B**) KEGG differential enrichment score chart. The horizontal axis represents the Differential Abundance (DA)-score value, DA-score = (number of upregulated substances—number of downregulated substances)/the total number of differential substances on the pathway. The vertical coordinate represents the metabolic pathway, and the size of the top point of the column indicates the number of differential metabolites enriched on the pathway. (**C**,**E**,**F**) ELISA detection of the key metabolite contents (PPARα, Taurine, 24(S)-hydroxycholesterol) in serum. (**D**) Relative expression level of the PPARα gene in the OP mice liver. OP group vs. Natto group (**, *p* < 0.01; ***, *p* < 0.001).

KEGG enrichment impact factor bubble plot (Figure 8A) and score plot (Figure 8B) revealed that the effects of differential metabolites between groups were primarily concentrated in pathways such as Primary bile acid biosynthesis, nuclear receptors PPAR signaling pathway, Vitamin B6 metabolism, Regulation of lipolysis in adipocytes, Caffeine metabolism, Arginine biosynthesis, and Galactose metabolism. Among these, the PPAR signaling pathway and Primary bile acid biosynthesis pathway were the main pathways upregulated by Natto. Therefore, we further measured the protein levels of PPARα, Taurine, and 24(S)-hydroxycholesterol in serum (Figure 8C,E,F) and the expression level of the PPARα gene in the liver (Figure 8D) using ELISA kits and qPCR methods. The results corroborated the trends observed in the metabolomics analysis. Based on these findings, we speculate that Natto may influence Primary bile acid biosynthesis by upregulating hepatic PPARα levels and affect downstream bile acid metabolism by increasing the protein levels of Taurine and 24(S)-hydroxycholesterol.

### 3.8. Correlation Analysis of Gut Microbiota with Bone Metabolism Indicators and the Mechanism Involved

#### 3.8.1. Correlation Analysis Between Gut Microbiota and Bone Metabolism Indicators and Mechanistic Insights

**Figure 9:**
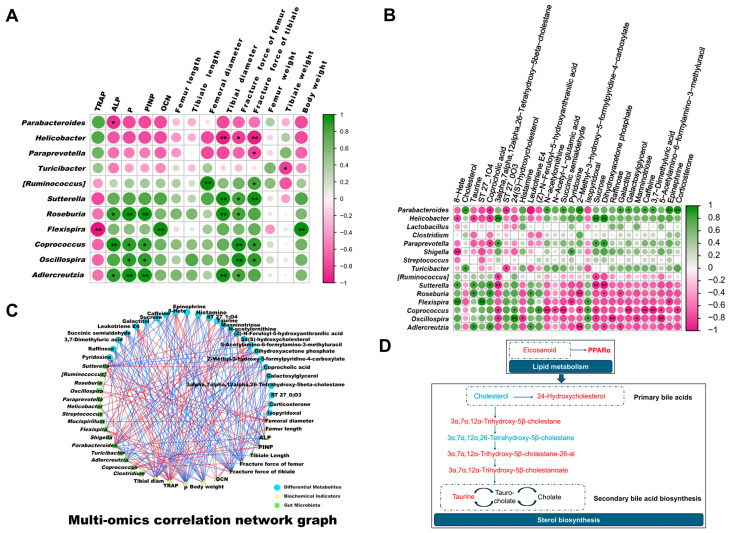
Spearman correlation analysis of the gut microbiota and serum metabolites, as well as physiological indicators in OP mice (**A**,**B**). Correlation Network diagram of gut microbiota, microbial metabolites, and biochemical indicators (**C**) and a schematic diagram of the action mechanism of natto (**D**). In (**A**,**B**), the listed items represent the intestinal microbiota, behavioral body indicators or differential metabolites. The green circles indicate positive correlations, while the pink circles indicate negative correlations. The larger the absolute value of the correlation, the larger the circular shape. *, ** respectively indicate significance *p* < 0.05 and *p* < 0.01. In (**C**), the number of connecting lines represents the strength of the correlation. In (**D**), Blue indicates substances with decreased levels, while red indicates substances with increased levels.

To better elucidate the anti-osteoporosis mechanism of natto as a fermented food, we further explored the relationship between gut microbiota and osteoporosis-related indicators through Spearman correlation analysis (Figure 9). The results (Figure 9A) showed that [*Ruminococcus*], *Sutterella*, *Roseburia*, *Flexispira*, *Coprococcus*, *Oscillospira*, and *Adlercreutzia* were positively correlated with body weight, tibia and femur dimensions, serum ALP, PINP, OCN, and P levels, and were significantly negatively correlated with TRAP levels. In contrast to the relationships observed for these bacterial genera, *Parabacteroides*, *Helicobacter*, and *Paraprevotella* were negatively correlated with fracture force, ALP, PINP, OCN, and P levels, and positively correlated with TRAP levels. Additionally, *Turicibacter*, which was significantly increased in the OP group and significantly decreased in the Natto and ND groups, showed a significant negative correlation with tibial weight (*p* < 0.05). Correlation analysis between these signature bacterial strains and serum differential metabolites revealed (Figure 9B) that *Parabacteroides*, *Helicobacter*, *Paraprevotella*, and *Shigella* were negatively correlated with metabolites such as 8-Hete, Taurine, Coprocholic acid, and ST 27_0;O3, and positively correlated with metabolites including Cholesterol, 3alpha,7alpha,12alpha,26-Tetrahydroxy-5beta-cholestane, Sucrose, and Dihydroxyacetone phosphate. Conversely, [*Ruminococcus*], *Sutterella*, *Roseburia*, *Flexispira*, *Coprococcus*, *Oscillospira*, and *Adlercreutzia* exhibited opposite correlations with the aforementioned metabolites. Furthermore, *Turicibacter* displayed a distinct metabolic correlation pattern compared to the other strains, showing a positive correlation with Cholesterol and a negative correlation with ST 27_0;O3. A subsequent correlation network analysis integrating the three datasets—16S rRNA gut microbiota data, serum metabolites, and bone metabolism indicators (Figure 9C)—revealed that *Sutterella*, *Parabacteroides*, *Adlercreutzia*, and *Coprococcus* were the representative bacterial genera most frequently associated with other data variables. This suggests that these bacterial strains may be key mediators linking natto to the regulation of serum metabolism and bone transformation via the “gut–bone” axis.

#### 3.8.2. Mechanistic Insights

**Figure A1:**
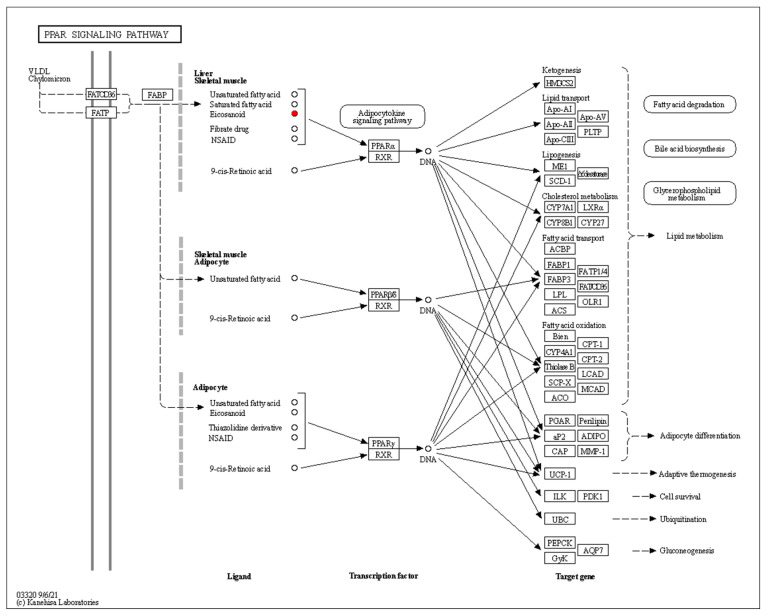
The KEGG map (03320) of PPAR signaling pathway. The red solid circles indicate the substances with increased relative content. The original signal pathway image can be downloaded at the following address (https://www.kegg.jp/kegg-bin/show_pathway?map03320/C14776%09red, accessed on 16 April 2026).

**Figure A2:**
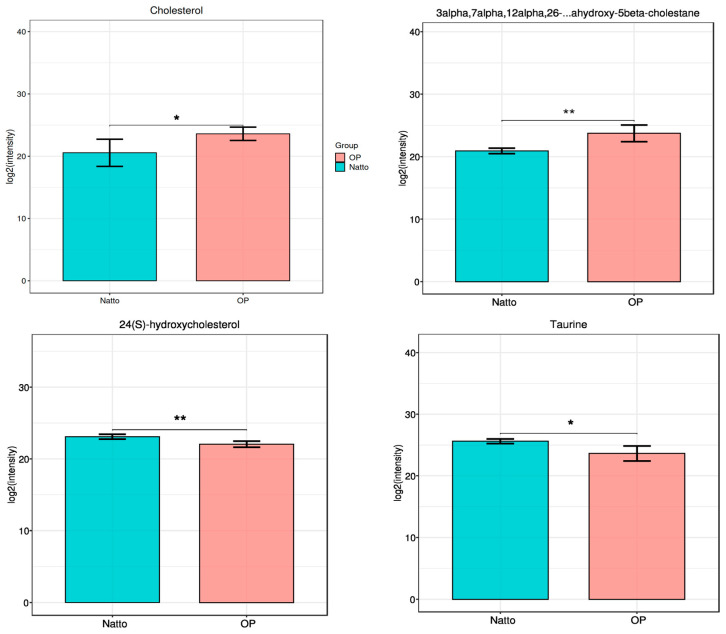
Relative content comparison of differential metabolites in the serum metabolomics. OP group vs. Natto group (*, *p* < 0.05; **, *p* < 0.01).

**Figure A3:**
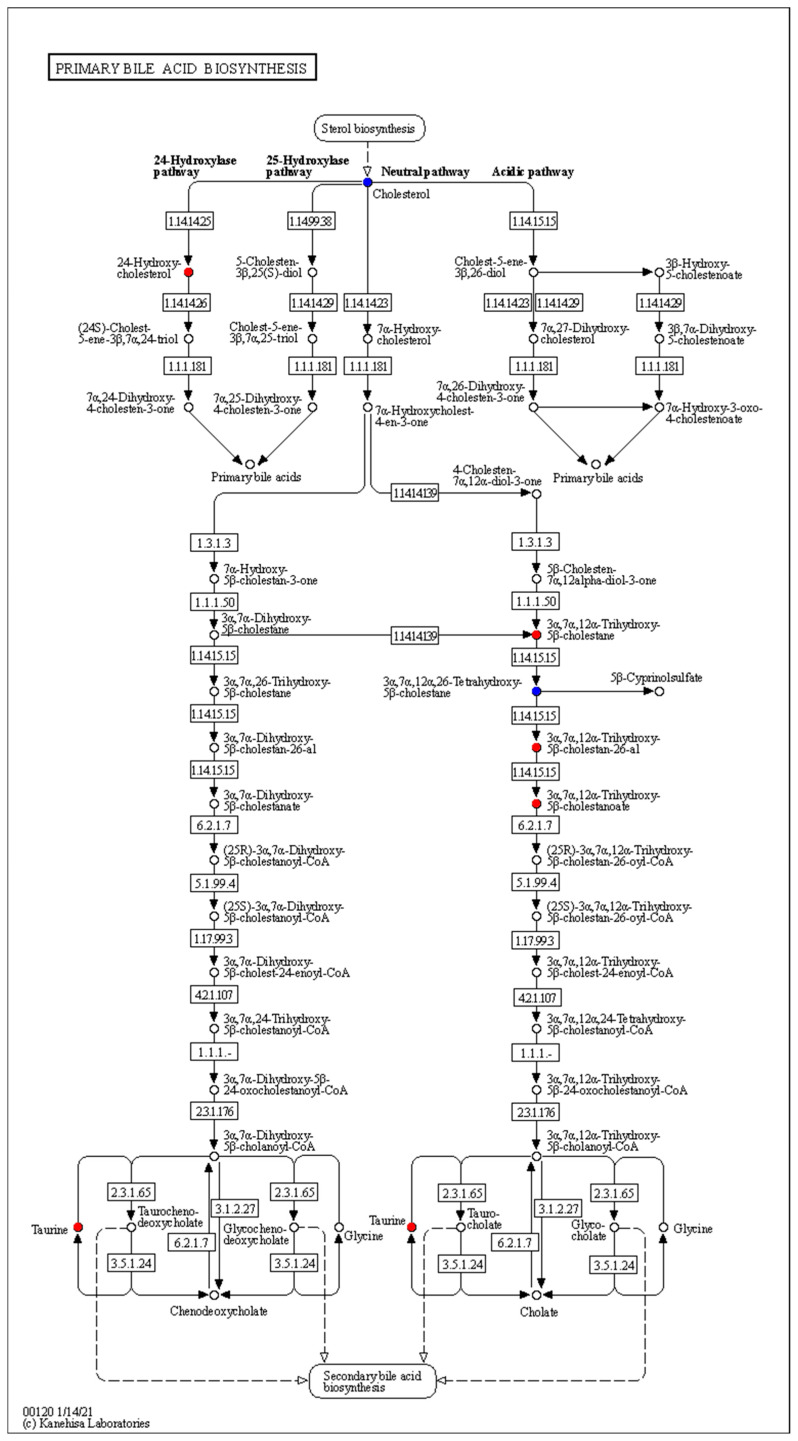
The KEGG map (00120) of Primary bile acid biosynthesis. The red solid circles indicate the substances with increased relative content, the blue solid circles indicate the substances with decreased relative content. The original signal pathway image can be downloaded at the following address (https://www.kegg.jp/kegg-bin/show_pathway?map00120/C00187%09blue/C00245%09red/C01301%09red/C04722%09red/C05446%09blue/C05454%09red/C13550%09red, accessed on 16 April 2026).

Integrating the hepatic qPCR and serum metabolomics results, it was observed that natto upregulated the expression of the PPARα gene in the liver of OP mice, increased serum Eicosanoid levels, and activated the PPARα pathway, which subsequently influenced downstream bile acid biosynthesis (Figure A1). During the process of bile acid biosynthesis, natto decreased the serum levels of Cholesterol and 3α,7α,12α,26-Tetrahydroxy-5beta-cholestane in OP mice, while increasing the levels of 24(S)-Hydroxycholesterol, 3α,7α,12α-Trihydroxy-5β-cholestane, 3α,7α,12α-Trihydroxy-5β-cholestane-26-al, 3α,7α,12α-Trihydroxy-5β-cholestanoate, and Taurine (Figure A2), ultimately affecting the anabolism of secondary bile acids (Figure A3). This suggests that the upregulation of hepatic and circulating PPARα expression may link the metabolic functions between the gut microbiota and organs by modulating the downstream bile acid pathway, thereby mediating the mechanism by which natto ameliorates retinoic acid-induced osteoporosis in mice through the “gut microbiota–PPAR/bile acid–OPG/RANKL signaling pathway” axis. To the best of our knowledge, this study is the first to reveal the role and mechanism of natto in preventing and treating secondary osteoporosis based on the “gut microbiota–PPAR/bile acid” pathway, providing a necessary theoretical foundation for the development of bone health food products from natto.

## 4. Discussion

Regular habitual consumption of natto has been reported to improve lumbar spine bone mineral density in perimenopausal women and total hip and femoral bone density in elderly Japanese men [23,24]. However, these findings are primarily derived from human cohort analyses with limited physiological indicators measured, and the significance often disappears after data correction, making it difficult to deeply investigate the mechanistic effects of natto. In recent years, the theory regarding the “gut–bone” axis has been gradually refined, but research on the anti-osteoporosis mechanism of natto and its impact on gut microbiota remains scarce. This study investigated the effects of a natto-containing diet on bone metabolism indicators, serum metabolic profiles, and gut microbiota structure in retinoic acid-induced osteoporotic mice, providing a necessary theoretical basis for improving osteoporosis through dietary interventions targeting the “gut–bone” axis.

Ovariectomy (OVX), drug-induction (such as glucocorticoids, retinoic acid), and immobilization model are three common methods for inducing osteoporosis. OVX is the gold standard for postmenopausal osteoporosis (primary osteoporosis), but it involves complex operational steps, severe injuries to experimental animals, and a long model establishment period (8–12 weeks). The glucocorticoid-induced model is highly correlated with the pathological mechanism of bone loss caused by long-term hormone therapy in clinical practice. Although this experimental period is shorter (2–4 weeks), high doses can lead to immunosuppression and significant individual differences. The immobilization model, although without drug interference, has a mild degree of bone loss and is prone to causing joint stiffness, and is not suitable for rapid evaluation of drug intervention effects. In contrast, the secondary osteoporosis model represented by retinoic acid has a simpler and faster modeling method. Retinoic acid is a human tumor suppressor, and long-term use of it can produce a series of side effects, including osteoporosis. The osteoporosis model established by intragastric administration of retinoic acid is highly similar to the human condition in terms of disease symptoms, bone tissue morphology, and response to estrogen, making it an ideal model for studying secondary osteoporosis [25,26]. This study showed that mice in the OP group, which received retinoic acid gavage, exhibited sparse trabecular bone structure, obstructed bone marrow cavities, significantly reduced fracture force, and significantly increased levels of TRAP, a marker of osteoclast formation, indicating successful modeling. Additionally, during the modeling period, mice in the OP group experienced severe hair loss, sluggish movement, and continuous weight loss, suggesting that retinoic acid caused certain gastrointestinal irritation in the mice. These changes in body weight and condition are similar to those observed in the previous study where rats were administered 75 mg/(kg·bw) retinoic acid daily for 2 weeks [26]. In contrast, mice in the Natto group consistently maintained higher body weight than the OP group, exhibited relatively intact trabecular bone structure, a larger bone collagen area, and significantly improved locomotor activity and hair regrowth. This indicates that a natto diet can significantly ameliorate drug-induced bone loss and sluggishness, even demonstrating an unexpected protective effect on hair. It is worth noting that excessive retinoic acid had a “transient” inhibitory effect on the food intake of mice (Table A1), meaning that this inhibitory effect disappeared rapidly after the retinoic acid dose was adjusted—the food intake of mice in the OP group and the Natto group recovered significantly during the final 10-day experiment period, and the levels were comparable to that of the control group at the end of the experiment. This indicates that the hunger effect caused by retinoic acid only temporarily affected the weight of the mice. With the rapid recovery of food intake and body weight, the effect of retinoic acid on interfering with bone metabolism persisted, while the natto diet produced a bone protection effect without relying on high food intake or high calorie intake (Table A2). Of course, further pair feeding or energy intake tests can more effectively refine the mechanism of natto’s effects, which is also an experimental design that we need to improve in our subsequent research. In conclusion, to the best of our knowledge, this study is the first to confirm the ameliorative effect of natto on secondary osteoporosis.

In this study, compared with the ND group, OP mice exhibited abnormally elevated serum TRAP and ALP levels, confirming that retinoic acid induced high bone turnover osteoporosis, which is consistent with previous research findings [27,28]. Natto significantly increased serum PINP, OCN, and P levels while decreasing serum TRAP and ALP levels in OP mice. Postmenopausal women often suffer from high-turnover osteoporosis [29]. The results of this study indirectly suggest that a natto-containing diet may improve osteoporosis in postmenopausal women by inhibiting high bone turnover. Substantial research evidence indicates that obesity is detrimental to bone health, with fat mass negatively correlated with bone mineral density [30]. Bone marrow mesenchymal stem cells can differentiate bidirectionally into adipocytes and OB. Excessive differentiation towards adipocytes leads to reduced osteoblast differentiation and mineralization, resulting in osteoporosis [31]. In this study, femoral pathological sections from OP mice showed extensive adipocytes, while sections from the Natto group showed significantly fewer adipocytes, suggesting that natto may promote the differentiation of bone marrow mesenchymal stem cells towards OB, thereby enhancing bone formation and mineralization. OPG is a soluble glycoprotein secreted by OB and bone marrow stromal cells that inhibits osteoclast formation and activity. RANKL is the only ligand capable of inducing osteoclast differentiation and exerting its biological activity; it binds to RANK on the osteoclast surface, thereby stimulating osteoclast activation, formation, and differentiation [22]. Since OPG can also bind to RANK, OPG, and RANKL exist in a competitive relationship, with increased OPG levels inhibiting osteoclast activation [22]. In this study, natto significantly increased femoral OPG protein expression and significantly decreased RANKL protein expression in OP mice, indicating that a natto diet can prevent and ameliorate osteoporosis by regulating the OPG/RANKL pathway. Furthermore, our team’s previous research has confirmed that natto and its fermenting strain, *Bacillus natto*, can improve obesity in mice by altering gut microbiota, inflammation levels, and intestinal barrier function [32]. It is hypothesized that the mechanism by which natto improves osteoporosis in this study may be related to its ability to regulate metabolic levels and intestinal function in obese mice.

Gut microbiota influence the connection between the intestine and bone metabolism through pathways involving the intestinal epithelial barrier, immune system, endocrine system, and microbial metabolites, operating via the “gut–bone” axis [17]. In numerous clinical studies, the gut microbiome has been demonstrated to exert profound effects on bone quantity, quality, and overall strength [33]. In the present study, the Natto group exhibited significantly downregulated abundance of *Prevotella*. Research has confirmed that presence of *Prevotella* is increased in patients with rheumatoid arthritis [34], suggesting that its elevation in the OP group might be associated with the pathogenesis of osteoporosis. A noteworthy observation in this study was that presence of *Helicobacter* was significantly increased in the OP group but significantly decreased in the Natto group. A study conducted in Japan found that *Helicobacter pylori* infection increases the risk of osteoporosis in women [35]. The results of our study suggest that a natto diet may antagonize *Helicobacter* infection. The abundance of *Shigella* was significantly elevated in the OP group and significantly reduced in the Natto group. Studies have confirmed that the abundance of *Shigella* is significantly increased in the gut of osteoporotic women and is associated with decreased systemic bone mineral density [36]. Other research has found that *Shigella* is significantly increased in the gut of mice with colitis complicated by osteoporosis, and inflammation is closely related to osteoclast differentiation and activity. Therefore, it is speculated that there is a certain connection between the intestinal environment, *Shigella* levels, and bone health [37]. These results also simultaneously imply the potential of natto in protecting gastrointestinal health against pathogenic bacterial infections. Notably, the high-abundance signature species in the OP group of this study was *Lactobacillus*, and the natto diet corrected this abnormal elevation of *Lactobacillus*. Research has found that excessive accumulation of *Lactobacillus* can lead to an increase in oxidative indicators, damage to intestinal epithelial cells, and retardation of organismal development [38], which might be one of the reasons that *Lactobacillus* exacerbates osteoporosis in mice.

Serum metabolomics results indicated that the regulatory effect of natto on metabolism in OP mice was primarily manifested in the Primary bile acid biosynthesis and PPAR signaling pathways. Further verification using liver and serum indicators showed that natto upregulated hepatic PPARα expression levels, activated the PPARα signaling pathway, and subsequently partially promoted bile acid biosynthesis. This process refines the mechanistic network of natto’s action—”gut microbiota–metabolites (bile acids)–bone turnover signaling pathway (OPG/RANKL)”—providing a new perspective for achieving multi-organ and multi-level improvement of bone metabolism through natto consumption.

Under both normal physiological and pathological conditions, bone tissue plays an important role in regulating systemic energy metabolism and adipogenesis [39]. PPARα protein is one of the key regulators of glucose and fatty acid metabolism. Research in recent years has shown that PPARα deficiency simultaneously downregulates pro-osteogenic signals of the Wingless-Type MMTV Integration Site Family (WNT), Transforming growth factor-beta (TGF-β), and Hippo pathways, indicating that PPARα directly regulates the bone cell-adipocyte metabolic axis, influencing the contribution of bone cells to overall energy metabolism [40,41]. Studies have found that PPARα deficiency can directly impact the expression level of the bile acid transporter Abcb11 in the liver and also reduces the mRNA levels of the phospholipid transporter ATP-binding cassette subfamily B member 4 (Abcb4, a phospholipid floppase) and the cholesterol transporters Abc transporter A1 (Abca1) and Abc transporter G5/G8 (Abcg5/Abcg8). PPARα regulates hepatic lipid homeostasis through its targeting of these Abc transporters, thereby influencing bile acid biosynthesis, transport, and secretion [42,43]. The present study found upregulation of PPARα mRNA in liver tissue and enhanced levels of circulating bile acid metabolites in the Natto group. These results suggest that natto may both directly influence osteogenic signaling pathways by acting on PPARα protein and indirectly affect bone metabolism and bone turnover through the PPARα–bile acid–FXR pathway.

The gut microbiota, which is linked to both PPARα and bile acid metabolism, likely represents a third pathway and target through which natto exerts its bone-protective effects. The gut microbiota hydrolyze conjugated bile acids synthesized in the liver (such as glycocholic acid and taurocholic acid) into free bile acids using unique enzymes like Bile Salt Hydrolase (BSH). This is considered the first step in bodily bile acid metabolism. Subsequently, certain bacterial groups (e.g., *Clostridium*) further convert primary bile acids into secondary bile acids (such as deoxycholic acid and lithocholic acid) through reactions like 7α-dehydroxylation, increasing the chemical diversity and hydrophobicity of bile acids [44]. Certain secondary bile acids (e.g., isolithocholic acid, 3-oxolithocholic acid) can act as ligands, directly binding to and activating PPARs, particularly the PPARα and PPARγ isoforms. Bile acids can also indirectly influence PPAR expression and activity by activating FXR. Conversely, PPAR activation can further regulate the composition and function of the gut microbiota by affecting the intestinal immune and barrier environment, or by secreting signaling molecules like bile acids and short-chain fatty acids, indirectly influencing microbial growth and metabolism [45,46]. Given its direct effects on both PPARα and the gut microbiota, natto is well-positioned to intervene in the aforementioned metabolic processes, effectively alleviating retinoic acid-induced osteoporosis in mice.

## 5. Conclusions

To our knowledge, this study is the first to systematically elucidate the molecular mechanism of natto in ameliorating retinoic acid-induced secondary osteoporosis and to construct a novel multi-pathway interaction network centered on the “gut–bone” axis. The results demonstrate that dietary intervention with natto not only directly corrected the high bone turnover state induced by retinoic acid in mice—reversing body weight loss, significantly increasing serum levels of bone formation markers (PINP, OCN) and P, and significantly decreasing levels of bone resorption markers (TRAP, ALP)—but also restored trabecular bone structure and increased bone collagen area at the tissue level, while inhibiting osteoclast activity through effective regulation of the OPG/RANKL signaling pathway. More significantly, this study revealed that natto indirectly exerts bone-protective effects by remodeling the gut microbiota structure and serum metabolic profile. Natto specifically downregulated the abnormal abundances of *Prevotella*, *Helicobacter*, *Shigella*, and *Lactobacillus* in the intestines of OP mice. These microbial changes were closely correlated with bone health indicators in the mice, suggesting that natto possesses the potential to regulate gut microbiota–metabolic homeostasis. In-depth serum metabolomics and hepatic gene expression analyses jointly indicated that the core anti-osteoporosis pathway of natto may involve activating the PPARα signaling pathway by modulating the gut microbiota, ultimately promoting primary bile acid biosynthesis. On one hand, natto directly regulates the “bone-adipose” metabolism and may promote osteogenic differentiation by upregulating hepatic PPARα expression. On the other hand, PPARα-driven changes in bile acid metabolism interact with the gut microbiota; nuclear receptors such as FXR and their downstream signaling molecules transmit regulatory signals remotely to the skeleton. We speculate that by modulating the gut microbiota, natto intervenes in this “PPARα-bile acid” axis, achieving systemic regulation of bone metabolism. Of course, this conclusion still requires further multi-angle experimental verification, such as antibiotic treatment, fecal microbiota transplantation, PPARα inhibition, or bile acid pathway inhibition. This is also the direction and theme of our subsequent research.

In summary, this study reveals the network mechanism underlying the anti-osteoporotic effects of natto, a fermented soybean product with a long history. Acting through the intestine as an entry point, natto influences circulating and hepatic PPARα expression and bile acid metabolism levels, thereby regulating the OPG/RANKL balance in bone tissue and cellular differentiation fate, ultimately achieving effective inhibition of bone loss. This conclusion not only provides a crucial mechanistic explanation for the positive correlation observed between natto intake and bone mineral density in clinical studies but also offers a strong theoretical basis and practical approach for dietary intervention strategies targeting the “gut–bone” axis to prevent and treat osteoporosis.

## Figures and Tables

**Table 1:** Effect of natto on the quality, length, and diameter of femoral and tibial bones in OP mice.

Group	Femoral Length/mm	Tibial Length/mm	Femoral Diameter/mm	Tibial Diameter/mm	Femoral Weight/g	Tibial Weight/g
ND	17.39 ± 0.33	20.35 ± 0.61	1.84 ± 0.08	1.46 ± 0.076	0.099 ± 0.003	0.079 ± 0.057
OP	15.94 ± 0.21 *	17.44 ± 0.76 *	1.60 ± 0.04 *	1.28 ± 0.039 *	0.080 ± 0.005 *	0.056 ± 0.005 *
Natto	17.39 ± 0.51 ^#^	18.61 ± 0.39	1.93 ± 0.01 ^#^	1.52 ± 0.01 ^#^	0.095 ± 0.003 ^#^	0.086 ± 0.003 ^#^

Note: A significant difference between the OP group and the ND control group (*, *p* < 0.05); the Natto group showed a significant difference compared with the OP group (#, *p* < 0.05).

**Table 2:** Effect of natto on the bone fracture force, serum ALP, TRAP, PINP, OCN, and P levels in mice.

Group	Femoral Fracture Force/g	Tibial Fracture Force/g	ALP/(ng/mL)	TRAP/(ng/mL)	PINP/(ng/mL)	OCN/(ng/mL)	P/(mM)
ND	1623.38 ± 129.93	1961.69 ± 241.23	2.10 ± 1.16	11.12 ± 0.76	2.65 ± 0.31	2.99 ± 0.29	0.145 ± 0.003
OP	757.68 ± 106.54 **	953.20 ± 105.00 **	6.84 ± 1.27 *	18.44 ± 1.46 *	0.66 ± 0.28 *	1.50 ± 0.053 *	0.114 ± 0.009 *
Natto	1390.57 ± 90.79 ^##^	1720.00 ± 39.92 ^##^	4.03 ± 0.53 ^#^	13.62 ± 1.49 ^#^	2.07 ± 0.52 ^#^	3.24 ± 0.70 ^#^	0.153 ± 0.121 ^#^

Note: ND group vs. OP group (*, *p* <0.05; **, *p* < 0.01), OP group vs. Natto group (#, *p* < 0.05; ##, *p* < 0.01).

## Data Availability

The original contributions presented in this study are included in the article. Further inquiries can be directed to the corresponding author.

## Appendix A

**Table A1:** The statistics of mouse food intake during the experiment (mean value within each group/g).

Date	Day1	Day2	Day3	Day4	Day5	Day6	Day7	Day8	Day9	Day10	Day11	Day12	Day13	Day14	Day15	Day16	Day17	Day18	Day19	Day20	Day21
ND	3.97	3.94	4.24	3.58	3.37	2.32	4.34	3.33	3.30	3.57	3.26	3.83	3.55	3.42	3.74	3.70	3.56	3.66	3.62	3.24	3.14
OP	3.48	3.78	4.14	2.39	1.66	1.02	0.86	1.29	1.48	1.77	2.05	2.85	3.39	3.51	3.84	3.29	3.21	3.39	3.34	4.26	3.53
Natto	4.17	3.92	2.61	1.95	1.21	0.88	1.02	2.93	2.37	3.16	3.09	3.16	3.70	3.62	4.19	3.81	3.27	3.21	4.22	4.37	4.14

**Table A2:** The major nutritional components and total energy of the experimental feed.

Nutritional Components	Crude Protein /100 g	Crude Fat /100 g	Crude Fiber /100 g	Total Energy/kcal/g
Basic feed	18.12 ± 1.05	4.26 ± 0.58	5.08 ± 0.43	3.88 ± 0.42
Natto feed	19.45 ± 1.12	4.02 ± 0.66	5.17 ± 0.39	3.83 ± 0.28
